# The potential role of cofilin-1 in promoting triple negative breast cancer (TNBC) metastasis via the extracellular vesicles (EVs)

**DOI:** 10.1016/j.tranon.2021.101247

**Published:** 2021-10-19

**Authors:** Jane Howard, Chia Yin Goh, Karolina Weiner Gorzel, Michaela Higgins, Amanda McCann

**Affiliations:** a UCD Conway Institute of Biomolecular and Biomedical Research, University College Dublin, Belfield, Dublin 4, Ireland; b UCD School of Medicine, College of Health and Agricultural Sciences (CHAS), University College Dublin, Belfield, Dublin 4, Ireland; c St Vincent's University Hospital (SVUH), Elm Park, Dublin 4, Ireland

**Keywords:** Triple negative breast cancer, Metastasis, Extracellular vesicles, Cofilin-1, Liquid Biopsy

## Abstract

Triple negative breast cancer (TNBC) is an aggressive cancer, particularly prone to metastasis and is associated with poor survival outcomes. The key to unravelling the aggressiveness of TNBC lies in decoding the mechanism by which it metastasises. Cofilin-1 is a well-studied member of the cofilin family, involved in actin depolymerisation. Studies have described the diverse roles of cofilin-1 including cell motility, apoptosis and lipid metabolism. Levels of cofilin-1 have been shown to be increased in many different types of malignant cells, with increased cofilin-1 protein levels associated with poor prognosis in patients with TNBC. Extracellular vesicles (EVs) are microvesicles typically around 100 nm in size, found in all biological fluids examined to date (Lötvall et al., 2014). Proteomic studies on extracellular vesicles (EVs) have shown that cofilin-1 is amongst the most frequently detected. Moreover, decreased levels of cofilin-1 potentially inhibit the release of EVs from cells. Additionally, Cofilin-1 is essential for the maturation of EVs and may also play a key role in the establishment of the *pre-metastatic niche,* thus promoting tumour cell migration. Further work into the exact mechanism by which cofilin-1 advances TNBC metastasis, may potentially prevent disease progression and improve outcomes for patients with TNBC.

## Highlights

• Triple negative breast cancer (TNBC) is a biological heterogeneous andn aggressive disease with a poor prognosis.
• Extracellular vesicles have been shown to play a role in mediating metastasis.
• Cofilin-1 has been detected in extracellular vesicles.
• Cofilin-1 is involved in promoting triple negative breast cancer metastasis.

## Graphical abstract

**Figure:**
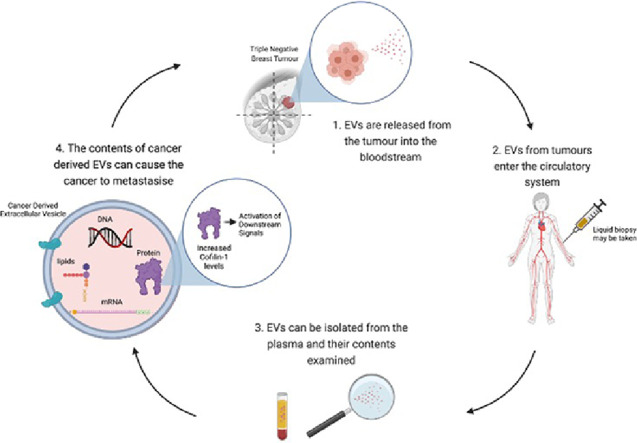

## Triple negative breast cancer (TNBC)

**Fig. 1:**
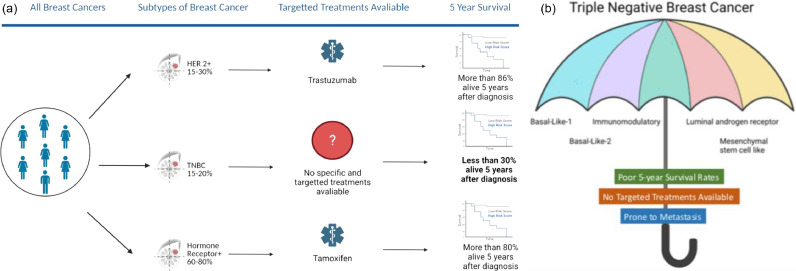
Subtypes of Breast Cancer, Targeted Treatments and Five-Year Survival. (a). TNBC accounts for approximately 10–15% of all breast cancer diagnoses. With 15–30% HER2 positive and 60–80% diagnosed as Hormone Receptor Positive. There is no specific and targeted treatment available for patients with TNBC, and less than 30% are still alive 5-years after diagnosis. (b). Triple Negative Breast Cancer is an umbrella term used to describe five known molecular subtypes of the disease. All cancers under the TNBC umbrella are prone to the development of metastasis with poor 5-year survival rates and lack of targeted treatments (created with Biorender.com).

Triple negative breast cancer (TNBC), is a particularly aggressive subtype of breast cancer which is Oestrogen Receptor negative (ER), Progesterone Receptor negative (PR) and does not overexpress the Human Epidermal Growth Factor Receptor 2 (HER2). TNBC accounts for 15–20% of breast cancers in Caucasian women (Fig. 1) and 20–40% of breast cancers diagnosed in African American women [54]. Recent studies have used gene expression profiles to further subtype breast cancers. Triple-negative breast cancers assessed in this way, can be found amongst 6 different molecular subtypes namely, *basal-like* 1, *basal-like* 2, immunomodulatory, mesenchymal stem cell-like and luminal androgen receptor [62]. While this further stratification has the potential to allow for personalised chemotherapeutic intervention, regardless of subtype, the individualised treatment options for patients remain limited. Triple negative breast tumours (TNBCs) are typically larger, of higher grade and more aggressive than hormone receptor positive breast tumours [24,86]. It has also been shown that TNBCs are more likely to present with lymph node metastasis at diagnosis, and typically spread to the lungs, liver and brain [24,86]. Despite initially responding to chemotherapy, women with TNBC tend to develop resistance to chemotherapeutic agents and subsequently metastasise more quickly than other subtypes of breast cancer [16].

### Current treatment and disease management

Treatment of breast cancer has been revolutionised by the personalised medicine era. The emergence of tamoxifen for the treatment of ER positive cancer, along with the development of trastuzumab for the treatment of breast cancers overexpressing HER2, has seen the number of breast cancer related deaths reduce significantly [35]. However, these therapies are not effective in TNBC, as this subtype of cancer lacks specific and targetable receptors. As a result, cytotoxic chemotherapy coupled with surgery is the most common treatment regimen for patients who have been diagnosed with TNBC [35,79]. Chemotherapeutics such as doxorubicin, carboplatin and cyclophosphamide are administered alone, or in combination as neoadjuvant or adjuvant therapy [35]. More recently, immune checkpoint inhibitors such as atezolizumab and pembrolizumab have shown efficacy when combined with chemotherapy, in both the primary and metastatic setting [63,64]. Additionally, adjuvant radiotherapy is often used for the treatment of localised TNBC, or palliation of symptoms from metastatic deposits [63]. Due to the innate heterogeneity of this breast cancer pathology, few specific molecular targets have been identified for patients with TNBC [37]. Despite extensive research in the area, it has been reported that fewer than 30% of patients with metastatic TNBC (mTNBC), are alive five years following diagnosis [1,2]. Conversely, it has also been reported that patients with residual disease following chemotherapy, typically have poor survival prospects in comparison to hormone receptor positive patients.

Despite best efforts to improve survival using cytotoxic chemotherapies, radiation therapies and surgery, overall survival for women who have been diagnosed with TNBC remains poor. The innate heterogeneity of disease presents substantial challenges in the areas of disease diagnosis, progression, and metastasis.

*Metastasis* is the general term used to describe the movement of primary cancers to surrounding tissue and distant organs. It is estimated that metastasis is responsible for 90% of cancer deaths [9]. For cancer to metastasise from the primary site to a secondary location such as the brain, cancer cells detach from the primary tumour, enter the systemic circulation or the lymphatic system and avoid death by evading the immune system. These cancer cells are then capable of relocating to a distant site in the body. The exact mechanisms that dictate the location of secondary tumours remain largely unknown. However, emerging evidence from the literature has suggested several molecular and cellular mechanisms that potentially play a role in organ-specific metastasis or *organotropism* [21]. The main cascade of events leading to metastasis includes detachment of tumour cells from the primary tumour, described as the epithelial to mesenchymal transition (EMT), an anchorage-independent survival, intravasation and dissemination into the bloodstream or lymphatic vessels, extravasation and finally establishment in the secondary site and sustained growth [88]. Metastatic cells are naturally capable of setting up a *niche* distal to the primary tumour which allows them to proliferate and promote angiogenesis [67].

Although the major steps of metastasis are well studied, the mechanisms by which metastatic cells arise from within populations of non-metastatic cell groups have only recently come to light [25,58]. EVs have been shown to play important roles in the development of the *pre-metastatic niche,* as they carry tissue specific blueprints and messages around the body [58]. EVs from cancer cells travel through the systemic circulation and are taken up by target cells distally, promoting a “homing” tumour microenvironment and subsequent cancer progression and metastasis [21]. Peinado et al., [58] described the role of EVs as “*education, progression and metastatic progression*”. A study by Hoshino et al., [25] demonstrated that EVs from tumour cells fuse preferentially with resident cells at their predicted destination. Additionally, they showed that it was possible to target specific EVs and decrease EV uptake and metastasis respectfully. Subsequent clinical data also showed that EV studies may be used to predict organ specific metastasis [25]. Therefore, given the prominent role of metastasis in TNBC disease progression, it has been hypothesised that the key to unravelling the aggressiveness of TNBC lies in decoding the mechanism by which TNBC promotes tumour metastasis and disease progression via extracellular vesicles (EVs) [27].

## The cofilin superfamily

**Fig. 2:**
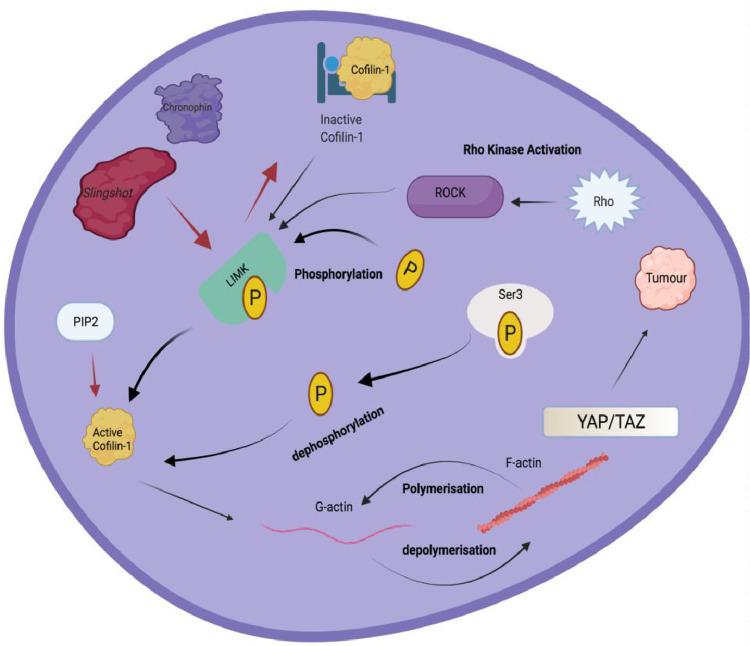
The diverse role of Cofilin-1 in the Human Cell. In the normal setting, dephosphorylation by the protein phosphatases, slingshot and chronophin of inactive cofilin, binds to F-actin or to actin monomers to induce rod assembly. Rho Kinase activation causes phosphorylation of LIM Kinase which activates cofilin-1, while dephosphorylation of SER3 also activates cofilin-1. Regulation of cofilin by PIP2, inhibits cofilin binding as they interact with the same region. Mechanical cues activate the transcriptional cofactors YAP and TAZ which have been implicated in cancer. Increased mechanical tension upon cofilin depletion promotes YAP and TAZ, enhancing transcription, proliferation and causing tumour growth in the cancer setting (created with Biorender.com).

Cofilin is a 19 kDa ubiquitous actin modulating protein encoded by the non-muscle isoform CFL1 (Gene ID: 1072). Cofilin is an important member of the actin depolymerising factor (ADF)/cofilin family which is comprised of cofilin-1 (CFL-1), cofilin-2 (CFL-2) and ADF in mammals. The ADF/cofilin family is a family of actin-binding proteins associated with the rapid depolymerisation of actin microfilaments that give actin its characteristic dynamic instability in almost all mammal cell types [78]. This dynamic instability is central to actin's role in muscle contraction, cell motility and transcription regulation (Fig. 2). Cofilin-1 is the most abundant isoform, predominantly expressed in non-muscle tissue. The ADF/cofilin family plays crucial roles in regulating actin dynamics by promoting actin treadmilling, driving membrane protrusion and cell motility [30].

Cofilins are of great physiological importance for cell movement *in vivo*. Their importance in embryonic development, health and disease has been studied extensively [6,^8^,13]. Despite their high degree of familial similarity at the amino acid level, cofilins have a varying affinity for actin [30]. Cofilin cascades play a huge role in homoeostasis, therefore, its levels are usually extremely tightly regulated. The regulation of cofilins and their emerging impact on cell motility has become of great interest as of late ([39,42]. Cofilin primarily influences actin dynamics in a two-step process by depolymerising F-actin and promoting its disassembly, leading to an increase in F-actin turnover. Additionally, it can sever F-actin so that it can be used in actin polymerisation [14]. The function of cofilins varies greatly and depends mainly on the supply of G-actin monomers available for actin polymerisation. Overall, the primary function of cofilin is its involvement in the regulation of actin assembly by severing actin filaments and increasing the number of filament ends from which monomers can be added or removed.

### The role of cofilin in actin regulation and locomotion

Although the members of the cofilin family share 80% homology, their affinity for binding actin varies greatly [34]. ADF and cofilin-1 are capable of binding and promoting steady-state F-actin (filamentous) disassembly. However, cofilin-2 is less efficient at the same task. This variation suggests a possible explanation for the lack of published research on cofilin-2 in the context of disease development [76]. The root of this *variation* is thought to be attributed to the fact that cofilin-1 and ADF originate from a location of higher actin turnover, as it is found in non-muscle tissue and the edges of motile or moving cells and cells undergoing mitosis ([6,46]), whereas the origin of cofilin-2 has been restricted to muscle tissue which does not require a high degree of actin turnover [23,76]. Controversially, a recent study by Chin et al., (2016) quantified the activities of cofilin-1, cofilin-2 and ADF using Total Internal Reflection Fluorescence (TIRF) microscopy. They found that cofilin-2 and ADF bind to actin and more readily sever actin filaments than cofilin-1. Interestingly, further studies to replicate this data are not available. Another study by Kremneva et al., [34], demonstrated that cofilin-2 has evolved specific and biochemical properties which allows it to control actin dynamics, potentially offering an association between cofilin-2 dysregulation and myopathies in mammals. Many tissues express all three isoforms of the cofilin family, with the cofilin family attracting most attention as a biomarker for cancers such as adenocarcinomas and osteosarcomas [85]. It has been hypothesised that each tissue type specifically regulates the expression of each isoform, depending on its location and functions to tightly regulate actin turnover and thus homoeostasis. Therefore, it may be suggested that the homoeostatic imbalance seen in cancer, can potentially be attributed to the cofilin family and dysregulated actin treadmilling.

Cofilins have been described as critical regulators of *actin-based* extension of cell membranes, known as membrane protrusions and the movement of cells from one place to another (locomotion) [8,46]. However, despite extensive research, it is conclusively unknown which member of the cofilin family regulates this cell movement [8,46]. A study by Tahamouni et al., [70] suggested that locomotion around the body was regulated by ADF and/or cofilin-1. Depletion of a single isoform of cofilin-1 showed changes in cell motility, changes in focal adhesion turnover and formation of abnormal actin structures. Cofilin-2 in contrast, was reported to be primarily localised between Z-disks in muscle sarcomeres, regulating the length of actin filaments. Cofilins are similar to actin in that they localise to the nucleus upon heat shock or dimethyl sulfoxide (DMSO) treatment. Increasing evidence has emerged, showing that cofilins can move into the nucleus and are involved in nuclear formation consisting of monomers, polymers, and rods. Nuclear actin has been reported to vary greatly. Specifically, nuclear actin polymers lack a filamentous structure, while actin rods are larger polymers that resemble cytoplasmic actin filaments [32]. However, the exact function of each isoform is not yet clear. The published reports rarely specify which isoform is being studied, or what specific role each isoform plays in the actin depolymerising activities. Moreover, it has been reported that the functions of the isoforms overlap greatly [30]. For this reason, the majority of research focuses on the mechanisms underlying the most abundant isoform, cofilin-1. For this review, where the specific isoform of the ADF/cofilin-1 family is mentioned, cofilin-1 or cofilin-2 will be used. Where the isoform is not specified, the overarching term of *cofilin* will be used.

### Cofilin regulation and dysregulation

The activity of cofilins is regulated by a variety of mechanisms including phosphorylation on residue Ser3 by LIM kinases and TES kinases which inhibits their interaction with actin [50]. LIM domain kinase 1 is a serine/threonine kinase that phosphorylates and leads to cofilin-1 inactivation, which results in actin polymerisation, and therefore promotes F-actin stability and maturation of functional invadopodia. Logically, LIM kinases (LIMKs) are required for invasion, as they promote the formation of invasive paths in collagen-rich environments during cancer cell migration [36]. In contrast, dephosphorylation of Ser3 leads to cofilin activation. The main protein phosphatases known to activate cofilin are slingshot [56] and chronophin [22]. Slingshot phosphatases can also regulate cofilin via dysregulation of LIMKs, resulting in inactivation of slingshots and thus decreasing levels of cofilins [66]. Another mechanism of cofilin regulation is binding to phosphatidylinositol 4,5-bisphosphate (PIP2) which acts as a competitive inhibitor as both proteins interact with actin at the same region. Hence, changes in levels of PIP2 can regulate and alter levels of cofilin [91]. Since EGF can promote the release of PIP2, EGF can affect mechanical interaction of LIM kinases with cofilin and also affect cell motility, protrusions and cell migration. Regulation of cofilin in this way is said to be independent of the LIMKs [65]. Furthermore, cofilin regulation can be affected by the intracellular pH as well as the sodium and hydrogen ion exchanger; NHE1. Regulation of cofilin *via* the PIP2 clustering is also pH sensitive, with higher pH inhibiting clustering of PIP2, therefore inhibiting membrane protrusions and motility [18,91]. There is some evidence to support the idea that cofilin appears to primarily bind to less- tense actin filaments and mediate their degradation, whereas filaments under tension are protected from cofilin-mediated fragmentation [73]. This mechano-regulation is important for the maturation of contractile stress fibres in cells [73].

Yeoh et al., [87] have also shown that pH affects actin severing and filament depolymerisation, with cofilin being much more potent at actin severing at higher pHs. Local variations in pH also influence the level of cofilin expression and cell motility. For example, in breast cancer patients, increased binding of cofilin to cortactin has been evident in promoting the formation of protrusions in aggressive breast cancers. Similarly, an increase in pH as a result of NHE1 mediation has been shown to result in a release of cofilin from cortactin, thus activating cofilin and resulting in cell invasion, migration and potentially promotes metastasis [47]. Despite a wealth of information, it is difficult to determine whether these roles are a direct result of the actin-severing activities, or whether these proteins have unappreciated functions as adaptor proteins [14,85]]. Evidence suggests that the deformed nuclear envelope seen in cancer cells facilitate successful cell migration through invasion through complex “*crowded environments”* which has been mediated by actin depolymerisation [15].

Disturbed activities of the ADF family and in particular, cofilin-1 has been shown to cause irreversible nuclear deformation [30]. This provides evidence that cofilin's actions are crucial, and dysregulation are frequently perturbed in the disease state, such as in the development of cancer.

### The role of cofilin in cellular proliferation and apoptosis

The cofilin family also plays an important role in the regulation of cell death or apoptosis, which is integral to the complex nature of cancer progression. Cofilin is capable of translocating to the mitochondria, which is crucial for the initiation of cell death [12],[41]. There are two major types of apoptosis. Intrinsic apoptosis occurs when a cell receives a signal to destroy itself, whereas extrinsic apoptosis occurs when a cell receives a signal to start apoptosis from another cell type. Cofilin is suggested to be involved in all stages of intrinsic apoptosis. Specifically, in human prostate cancer, TGFβ induced apoptosis requires mitochondrial translocation of cofilin. Moreover, it has been suggested that cofilin-1 is involved in the initiation of apoptosis potentially with other actin proteins [51]. In addition to this role in the early stages of apoptosis, cofilin may also be involved in the regulation of apoptosis-associated morphologies during the later stages such as in apoptosis-associated bleb formation [51]. Additional evidence has emerged, describing the association between apoptosis induced cancer cell blebbing, and extracellular vesicle (EV) release. It is thought that these mechanisms favour dissemination, cell-to-cell communication, and growth of cancer cells in the metastatic site [7]. The cofilin family also play a role in nuclear actin transport, transcription, nuclear architecture, and lipid metabolism. Specifically, cofilin-1, is an important mediator of cell movement by controlling actin dynamics during cell protrusion. The activity level of cofilin-1 is affected by expression level, phosphorylation level, pH and subcellular localisation [82]. Therefore, these factors may correlate closely with enhanced, cell survival, metastasis, invasion and tumour development [82].

Another important and complementary role for cofilins, is the regulation of cell proliferation. Cofilin has been shown to mediate actin cytoskeleton remodelling [70]. Mechanical cues activate the transcriptional cofactors YAP and TAZ. These YAP and TAZ pathways are crucial for cell proliferation during development and have been implicated in various diseases such as cancer. Increased mechanical tension upon cofilin depletion promotes YAP and TAZ, enhancing transcription and proliferation [5]. Conversely, it has been shown that cytoskeleton remodelling, or release of tension inhibits proliferation. Crosstalk between the YAP and TAZ pathways are the main driver of a subset of aggressive cancer such as uveal and skin melanomas [5]. Cells from these melanoma pathologies, show reduced cofilin activity, promoting actin cytoskeleton stability and activation of YAP. YAP activation is sensitive to inhibition of either contractility or actin polymerisation [17]. Based on the research investigating the role of cofilin in apoptosis and also cellular proliferation, it is clear that the role of cofilin in specific cancers forms an interesting and warranted topic for investigation.

## The role of cofilin-1 in cancer and metastasis

Evidence suggests that cofilin expression is altered in malignant cells. Specifically, cofilin-1 mRNA has been reported to be increased in various malignant cells such as adenocarcinomas, osteosarcoma, and lymphoid tissue, in comparison to control tissue [14]. Increased cofilin expression has also been shown to be associated with poor prognosis in human pulmonary adenocarcinoma, gastric cancer, epithelial ovarian cancer, and gall bladder carcinoma [[55],[59],[85]]. Recent advances have suggested a correlation between increased dephosphorylated cofilin expression and poor prognosis in a mixed cohort of triple negative and hormone receptor positive breast cancer patients [48]. Therefore, there is good evidence suggesting that dysregulation of the normal function of cofilin-1, is involved in the formation of the malignant phenotype. Also, reports have shown that cofilin is directly associated with invasion, intravasation and metastasis of mammary tumours [81]. However, there is limited evidence implicating a correlation between dysregulation of cofilin expression in breast cancer and its effect on prognosis at present in the literature [81].

As discussed previously, cancer cell progression and/or metastasis relies on the movement of cancer cells to another part of the body by cell migration. Importantly, the role of cofilin in cell proliferation suggests that cofilin is a key player in cancer cell growth and subsequent tumour enlargement [80]. In response to chemical signals in the body, cancer cells form membrane protrusions and subsequent actin filaments to initiate the migration process. The actin framework is widely accepted as the driver that regulates the assembly and disassembly of actin filaments and the dynamic behaviour of the actin cytoskeleton via actin treadmilling [80]. It is known that cofilin plays a crucial role in cytoskeleton formation via actin treadmilling by inducing lamellipodia formation which is involved in determining cell movement, a mechanism known to be implicated in cancers [81]. SiRNA depletion of cofilin in colorectal cancer cells have been shown to inhibit cell motility, stability of lamellipodia and cell invasion [84]. It has therefore been hypothesised, that malignant cells display excessive protrusion activity due to aberrant activation of signalling pathways that regulate the actin cytoskeletal arrangement [81].

Invadopodia, are matrix protrusions with a matrix degradation activity formed by invasive cancer cells [10]. Invadopodia, extend from a cell into the extracellular matrix, thus becoming motile. These invadopodia are enriched with actin filaments, actin-binding proteins and adhesion proteins forming many hypotheses around the mediation of this response [13]. Cofilin is a critical regulator of lamellipodia formation as well as actin dynamics [13]. Cofilin stimulates lamellipodia protrusion and cell migration. However, its function in the invadopodium has not been extensively studied. A study by Yamaguchi et al., [84] showed that EGF and EGF receptor signalling are responsible for invadopodia formation in highly metastatic adenocarcinoma cells which resulted in the formation of actin dot-like structures observed, possibly associated with cofilin levels. Specifically, high cofilin expression were observed at the lamellipodia and elevated expression has also been seen at the invadopodia. Interestingly, the invadopodia were shortened in cofilin siRNA-treated cells compared to control cells. Cofilin knockdown cells showed a compromised ability to invade and degrade actin matrices than control cells. The life-cycle of these treated cells were also shortened by cofilin siRNA knockdown compared to untreated cells. Therefore, it these authors concluded that cofilin is involved in adenocarcinoma cell migration and invasion via the invadopodium which mediates extracellular matrix degradation forming major protrusion structures which are formed by metastatic cancer cells in the 3D environment [84]. Additionally, it was concluded that using treatments to target cofilin may reduce migration and invasion, thus reducing the development of metastasis [84].

Another study looking at human bladder cancer cell proliferation, migration and invasion, showed that increased miR-182–5p could potentially inhibit tumour growth by repressing cofilin-1 expression [78]. Specifically, miR-182–5p is considered a tumour suppressor in renal cell cancer, non-small cell lung cancer (Li et al., 2018), osteosarcoma [90] and glioblastoma [33], while it is considered an oncogene in breast cancer [42], ovarian cancer [83] and prostate cancer [77]. Wang et al., [78] showed that cofilin-1 is a direct target of miR-182–5p in human bladder cancer and that cofilin-1 promotes tumour progression through a miR-182–5p/cofilin regulating axis. Loss of miR-182–5p was shown to promote cofilin-1 expression and subsequent tumourigenesis, migration and invasion. Therefore, loss of miR-182–5p in bladder cancer and subsequent promotion of cofilin expression presents a potential diagnostic and targeted therapy for bladder cancer [78].

A study by Maimaiti et al., [49] investigated the association between cofilin-1 and breast cancer prognosis to establish the role of cofilin in invasive breast cancer and correlated the results with increased expression and patient clinicopathological findings. They analysed the expression of cofilin-1 in tissue microarrays of 310 patients with various subtypes of breast cancer using immunohistochemistry. Increased cofilin expression was not observed to be correlated with oestrogen or progesterone receptor expression, tumour size or lymph node status. However, the study did suggest that increased cofilin is associated with significantly poorer outcome (*p* = 0.002) and that it is a potential prognostic indicator in breast cancer. Maimaiti et al., [48] used Kaplan Meier Analysis and the Breslow test to determine the effect of cofilin on overall survival. It was found that increased cofilin scores were associated with HER2 positivity, as well as increased expression of Ki-67 associated with increased proliferative potential. No association was observed between cofilin levels and age, tumour size, lymph node metastasis, oestrogen or progesterone receptor positivity. Kaplan Meier analysis demonstrated that the difference in overall survival between high and low expression of cofilin may be illustrated by the hazard ratio of 2.22, concluding that the activity and outputs of the cofilin pathway are increased in cancer cells ([80]; Ono, 2003) contributing to initial cell transformation [20] and increased cell motility during metastasis and cell division [71]. It was also concluded that increased cofilin activity was associated with poor prognosis in HER2 positive and TNBC subtypes which are inherently more aggressive. There is no evidence for the correlation between cofilin expression and tumour stages [49], however, increased cofilin levels were associated with shorter overall survival. The increased cofilin levels seen in patients with aggressive tumours may be driving the excessive migration of cancer cells. Therefore, cofilin targeting represents a potential therapeutic target for inhibiting cancer progression.

MicroRNAs (miRNAs) are endogenous RNAs capable of suppressing target gene mRNA translation. miRNAs play crucial roles in cell proliferation, cell differentiation and cell death where recent studies have pointed at the role of miRNAs in human cancers by acting as tumour suppressors or oncogenes. While some miRNAs are not correlated with tumourigenesis, some specific miRNAs may have a close correlation. The role of cofilin in TNBC has been further confirmed by Li et al., [39]. They found that microRNAs; miR-200b-3p and miR-429–5p suppress proliferation, migration, and invasion in TNBC cell lines, via inactivation of the LIMK1/CFL1 pathway, therefore acting as tumour suppressors, and suggesting that blocking this pathway has a potential therapeutic benefit when treating TNBC. The LIM domain is a highly conserved cysteine-rich domain that participates in protein-protein interactions. Cofilin-1 is one of the most studied LIM domain family targets.

In 2018, Li et al., [40] investigated the effect of miR-519–3p on the proliferation and motility of MDA-MB-231 cells. This paper reported that miR-519–3p expression was also associated with cancer metastasis and clinical staging [43]. Additionally, miR-519–3p was also shown to target the LIMK/CFL1 pathway. Via phosphorylation of cofilin-1, LIMK was shown to suppress actin severing activity therefore decreasing actin cytoskeleton organisation. As LIMK has been shown to be an essential molecule for migration and invasion, by stimulating cancer cells to form an invasive pathology, it has been suggested that LINK may be a potential strategy for treating progressive, invasive TNBC.

Although LIMK2 has been implicated in several cancer types, the role of LIMK2 in breast cancer is not fully understood. Malvi et al., [50] have shown that LIMK2 is overexpressed in TNBC compared to other breast cancer subtypes. LIMK2 overexpression was also associated with increased cancer incidence and metastasis. Therefore, modulating the LIMK/CFL1 pathway offers potential for the personalised treatment of TNBC. Another study by Liu et al., [44] examined the role of the microRNA-342–3p in TNBC and its role as a tumour suppressor via modulation of cofilin-1. Cofilin-1 was found to be upregulated in breast cancer tissues and cell lines. Interestingly, overexpression of miR-342 caused significant depletion of cofilin-1 in TNBC cell lines along with decreased cell proliferation, colony formation and migration. It was demonstrated that miR-342 inhibits the proliferation and migration of the triple negative breast cancer cell invasion by targeting cofilin-1 and promoting apoptosis which identifies miR-342 as a novel therapeutic target in breast cancer. Liu et al., also focused on the potential role of cofilin-1 in cell cycle arrest [44]. Finally, the role of HDAC6 in reducing TNBC migration has been studied by Hseih et al., [28]. As discussed, cofilin initiates actin polymerisation and directs cell migration which in turn promotes breast cancer metastasis. Phosphorylation of cofilin tightly regulates the severing and depolymerising of actin. HDAC6 inhibition was shown to cause cofilin phosphorylation and subsequent inhibition of actin polymerisation [3]. Therefore showing that a HDAC 6 inhibitor suppresses TNBC metastasis by inhibiting HDAC6 activity and inhibiting the cofilin/F-actin pathway but also inhibiting cortactin/F-actin binding and thus impairing cell motility and providing a potential therapeutic option for TNBC treatment [3],[28].

## Cofilin-1 delivered by extracellular vesicles promotes TNBC metastasis

Extracellular vesicles (EVs) are microvesicles typically around 100 nm in size found in all biological fluids examined to date [45]. The concept of EVs was first coined by Rose Johnstone [29] and since then, significant efforts have followed to develop the field of EVs [1,^29^,45]. While various subtypes of EVs have been classified based on physical properties including exomere, exo-small (exo-S) and exo-large (exo-L), recent studies have shown that it is impossible to strictly distinguish between these populations [45]. For this reason, the term extracellular vesicle has been deemed the appropriate term to describe these nanoparticles. EVs are highly representative of their cells of origin and can contain components of a cell including and not limited to; DNA, RNA, lipids, metabolites and surface proteins. The specific role of EVs has been of significant interest in the current literature. . Specifically, it has been reported that EVs may play a role in (i) metastasis, in the context of the development of pre-metastatic niches, (ii) the removal of excess cell constituents, (iii) the maintainence of cellular homoeostasis and/or (iv) playing a role in cellular communication. Technological advances are likely to yield further, more detailed information regarding the heterogeneity of EVs and their function biologically. It has been suggested that EVs associated with cancer progression deliver proteins, metabolites, and nucleic acids to recipient cells to alter/enhance the cells’ biological response [52]. Studies have also shown the ability of EVs to deliver therapeutic agents to their delivery target. For example, EVs may be capable of acting as a vehicle to transport chemotherapy to cancer sites. As EVs have been harvested from all biological fluids including blood, urine, cerebrospinal fluid and saliva, these complex vesicles are readily available via liquid biopsies [45]. EV based liquid biopsies also highlight their potential use as a biomarker in patients with cancer and other aggressive diseases. EVs are of particular interest in biology, as their formation involves a distinct intracellular regulatory process that likely determines their composition and function once secreted into the extracellular space. Due to their endocytic origin, EVs also carry valuable information from their cells of origin. Studies examining the RNA, DNA, protein, lipid, and metabolite contents of the EVs have emerged as the contents have been implicated in the development of drug resistance, cancer progression and metastasis [52].

**Fig. 3:**
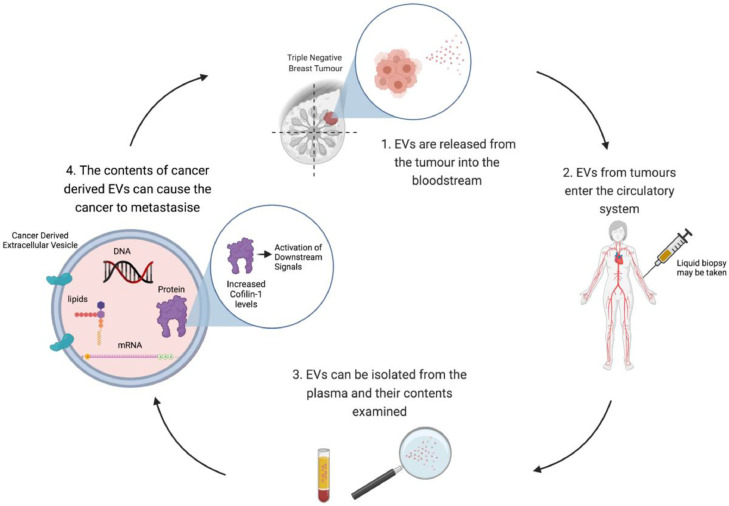
EVs released from triple negative breast tumours enter the circulatory system and may be detected in the plasma or other bodily fluids via liquid biopsy. EVs may then be isolated from the plasma and their contents examined. Contents include DNA, mRNA, lipids and proteins. The contents of cancer derived EVs have been shown to play a role in pre-metastatic niche formation [58]. Cofilin-1 may be packaged inside the EVs, therefore causing downstream signalling, contributing to cancer progression and metastasis (created with Biorender.com).

In each liquid biopsy type, subpopulations of EVs are present and display different amounts of cellular content. Proteomic analyses of EVs have revealed marker heterogeneity of EVs which have been shown to suggest a protein sorting mechanism associated with EV biogenesis and/or content loading. Based on this heterogeneity, the effect of EVs on recipient cells can be drastically different, depending on their content. In one liquid biopsy, groups of EVs may induce cell survival, another may induce apoptosis or immunomodulation, adding to the complexity and the innate heterogeneity of EV populations [61]. Additionally, the heterogeneity of EVs seen in fluids, such as plasma, stems from the location of origin and exposure to advantageous tropisms or uptake to specific cell types. A study by Hoshino et al., [26] investigated the proteomic profile of extracellular vesicles and particles in 426 human samples. To confirm that EVs are ideal diagnostic tools, they showed that there were specific proteins capable of distinguishing tumour tissue from normal tissue (Fig. 3). They also developed a panel of tumour-type specific proteins capable of classifying tumours of *unknown primary origin*. Therefore, Hoshino et al., [26] showed that EV proteins serve as reliable biomarkers for cancer detection and determining cancer [26].

Research has shown that plasma from patients with breast cancer contains substantially more EVs than age-matched controls [68], with further evidence demonstrating a higher concentration of EVs in the plasma of women with TNBC compared to healthy controls (*p* = 0.002) [[19],[68]]. It is thought that the increase in the shedding of EVs into the circulation is a general phenotype of cancer, and that cancer cells may be using their EV release as a mechanism of mediating metastasis [19]. EVs secreted by cancer cells have been shown to display differential protein [26] and miRNA [44] profiles compared to normal cells, potentially providing important information about the tumour from which they originate [21]. EVs have also been shown to be integral to the senescence-associated secretory phenotype (SASP) which has been shown to promote tumour growth. A study by Kavanagh et al., [31] showed that cancer cells that have undergone therapeutic induced senescence release significantly higher concentrations of EVs compared to non-senescent cells. These EVs released from senescent cells also showed an increase in proteins involved with cell proliferation, ATP depletion and SASP factors, which potentially promote tumour survival and migration. EVs are described as biological messengers within an organism.

It has been shown that EVs are capable of leading cancer progression and metastasis by transferring biological traits from their tumours of origin. EVs are described as biological messengers of cells, Zhang et al., [89] has shown that mRNA transported by EVs may potentially be translated into proteins once taken up by the recipient cell. Therefore, providing evidence that this mechanism of cellular communication may contribute to tumour microenvironment interactions, tumour progression and metastasis. A study by O'Brien et al., [57] cultured TNBC cell line Hs578T and its isogenic subclone. Co-culture experiments of Hs578T cells with EVs showed that EVs from the isogenic clone caused significantly increased growth, proliferation rate and migration of the cells confirming that EVs from TNBC cell lines can increase the invasiveness of the recipient cell. Further research by O'Brien et al., [57] isolated EVs from the serum of patients with TNBC. The EVs caused greater invasion of TNBC cells compared to age-matched controls, further confirming the importance of EVs in TNBC metastasis [57].

The "seed and soil hypothesis" supports the idea that a *pre-metastatic niche* is required for tumour cells to grow in secondary sites, and that EVs play an immensely important role in *nourishing* this pre-metastatic niche [60]. As EVs migrate through the circulation, they interact with and are taken up by recipient cells. EVs have been shown to be involved in organotropism; the organ-specific movement of cancer cells causing metastasis [72]. Hoshino et al., [25] examined EVs from the MDA-MB-231 TNBC cell line, known to metastasise to the lung. Specific integrins which facilitate lung tropism were identified within the EVs. Integrins playing a role in brain metastasis have also been identified in EVs from primary breast cancer [25]. These studies highlight the potential importance of studying EV signatures to predict the site of metastasis ahead of clinical imaging. As EVs can transport biological material around the body in patients with TNBC, research has also shown that EVs may also be capable of conferring drug resistance in TNBC. Valadi et al., [75], have shown that EVs from chemo resistant TNBC cells are able to induce proliferation and confer resistance in non-malignant breast epithelial cells, suggesting that chemoresistance can be transferred between cells via EVs [75]. TNBC cells have also been shown to expel chemotherapeutic agents via EVs as a potential mechanism to enhance drug resistance. Kavanagh et al., [31] showed that EVs released from chemo-resistant, senescent cells had significantly higher concentrations of chemotherapy compared to EVs from control cells.

EVs represent novel therapeutic targets in TNBC, and the EV pathways may be targeted at various stages such as biogenesis, release, and uptake. There is evidence to suggest that EVs may be the perpetrators of TNBC progression and metastasis. However, the exact underlying mechanisms are yet to be elucidated. Liquid biopsy studies of TNBC derived EVs, may help to identify the pre-metastatic niche, aid the diagnosis of cancer, and prevent chemoresistance. Therefore, detailed investigations into the contents of EVs is warranted to enrich our understanding of the role of EVs in TNBC, and to improve treatment and outcomes for patients with TNBC.

The cofilin family is readily detected in extracellular vesicles, where it is amongst the top 100 proteins detected in EVs to date. However, detailed studies into the exact role of cofilin-1 in cancer is still somewhat limited. Recent studies have emerged regarding the potential role of cofilin-1 in promoting cancer growth and metastasis (Table 1.) via EVs. A study by Amorim et al., [4] investigated the effect of a single oncogene on the EVs proteome. A non-cancerous (HB4a) and a HER2 positive breast cancer cell line (HB4a clone) were cultured and EVs collected via ultracentrifugation. Proteins were digested and subjected to LC-MS/MS. It was found that proteins capable of inducing malignant transformation such as cofilin-1 were overexpressed in the EVs from the breast cancer cell line compared to the non-cancerous control cell line. Cofilin was said to be involved in activating Rho-associated protein kinase which leads to LIM kinase phosphorylation of cofilin, preventing it from physiologically severing actin, resulting in extended actin fibres.

**Table 1:** The role of Cofilin-1 in Cancer and Metastasis A summary of cancer studies, cofilin status and conclusions regarding cancer prognosis. (Ordered as they appear in the text).

Reference	Cancer Type	Cofilin Status	Outcome
[59]	Pulmonary Adenocarcinoma	Increased cofilin protein in severe disease	Five-year survival rate for strongly positive group very poor (0%)
[55]	Advanced Epithelial Ovarian Cancer	Increased cofilin expression in severe disease	Cofilin-1 positive patients showed decreased progression free survival (*p* = 0.039)
[85]	Human Bladder Cancer	Increased cofilin expression and phosphorylation in invasive disease	–
[48]	Human Breast Cancer	Elevated cofilin expression	Poor clinical and survival outcomes
[84]	Highly metastatic Adenocarcinoma	Increased cofilin expression	–
[78]	Human Bladder Cancer	Increased cofilin-1 expression	Promotes tumour progression, invasion, and metastasis
[49]	Human Breast Cancer	Increased cofilin-1 expression	Shorter overall survival (*p* = 0.002)
[39]	Triple Negative Breast Cancer	Inactivation of CFL1/LIMK pathway	Decrease invasion and metastasis
[44]	Triple Negative Breast Cancer	Cofilin-1 upregulated	Increased cell proliferation and migration
[28]	Breast Cancer	Inhibition of cofilin pathway	Suppress TNBC metastasis
[4]	Breast Cancer EVs	Cofilin-1 overexpression	
[11]	Hepatocellular Carcinoma	Higher cofilin-1 concentrations in EVs	Advanced tumour stage, poor disease-free survival, poor overall survival
[69]	Gastric Cancer Cells	–	EVs from chemotherapy resistant cells translocated cofilin-1 to the mitochondria
[53]]	Hepatocellular Carcinoma cell EVs	Decreased miR-200–3p increased LIMK/CFL1 activation	Promote angiogenesis and cancer progression
[74]	Breast Cancer EVs	Downregulation of phosphorylated cofilin	Promoting brain metastasis

A recent study by Moh-Moh-Aung published in 2020 [53], looked at the effect of decreased levels of miR-200b-3p on hepatocellular carcinoma (HCC) cells. When they examined whether EVs isolated from cancer cells could transfer miR-200b-3p to endothelial cells, they found that miR-200b-3p is transferred via EVs from hepatocytes to endothelial cells, resulting in suppression of endothelial ERG expression and increased angiogenesis of tumour tissues. They showed that EVs isolated from HCC cells display decreased miR-200b-3p which was seen to promote angiogenesis and cancer progression in HCC tissues. This cancer growth or angiogenesis may be mediated by decreased levels of miR-200b-3p seen in HCC cell lines which have been shown to elicit tumour suppressor qualities in the highly aggressive HCC cell lines. Also, a decreased level of miR-200b-3p in EVs of aggressive HCC cells was shown to promote angiogenesis. Therefore, it might be suggested that decreased miR-200b-3p in the EVs of HCC cells causes angiogenesis via the LIMK1/CFL1 pathway. This confirms the need to further establish the role of miR-200b-3p in EVs as well as its association with the cofilin pathway in HCC metastasis. In a study by Cho et al., [11], protein markers of HCC derived EVs were evaluated from human HCC cell lines and an immortalised normal hepatocyte cell line. Proteomic analysis of HCC derived EVs revealed that 15 proteins were markedly overexpressed, and their clinical relevance was then tested on public RNA-sequencing datasets [11]. Amongst them, cofilin-1 was selected as a candidate biomarker. Higher cofilin-1 concentrations were associated with advanced tumour stage, poor disease free survival and poor overall survival [11].

In order to investigate the effect of role of cofilin-1 in mediating cancer metastasis via the EVs, Sun et al., [69] investigated the effect of treating chemotherapy sensitive cells with EVs from chemotherapy resistant cells. EVs were isolated from a cisplatin resistant gastric cancer cell line, they were then co-cultured with cisplatin sensitive cells. The EVs were readily taken up by the cisplatin sensitive cell and thus triggered a phenotype of chemoresistance in the receptor cells [69]. A further mechanism study demonstrated that EVs from cisplatin resistant gastric cancer cells communicate with cisplatin sensitive cells by translocating cofilin-1 into the mitochondria. Therefore, it was concluded that targeting EVs in cisplatin-resistant gastric cancer cells may provide a promising strategy to target cofilin translocation and overcome cisplatin resistance in gastric cancer.

According to Li et al. [38], microvesicles have been shown to decorate the surfaces of highly metastatic MDA-MB-231 TNBC cell lines. It has also been shown that the incubation of normal cells with EVs from highly metastatic cells resulted in the transformation of recipient fibroblasts. When fibroblasts were exposed to EVs from MDA-MB-231 cells, metastatic breast tumours formed in 3 out of 6 mice. Further analysis showed that the tumour masses were due to the EV-stimulated growth. The mechanism was unknown. Therefore, the role of Ras, Rac, Rho and Cdc42 were investigated as these GTPases are known for their ability to recognise the actin cytoskeleton. Results showed that the RhoA status of these cells affected their ability to produce microvesicles. Downstream of RhoA, LIM-kinase (LIMK) and myosin light chain phosphatase, are known regulators of actin cytoskeletal dynamics. Activated ROCK phosphorylates LIMK, stimulating its kinase activity and enabling it to phosphorylate Ser3 on cofilin, which prevents cofilin from severing actin filaments and prolongs the extension of actin fibres. They also investigated the role of cofilin, the major downstream effector of LIMK in EV formation. It was found that the expression of a cofilin S3a mutant significantly reduced the number of microvesicles isolated from the cells. The effectiveness of LIMK knockdowns was inversely correlated with the number of microvesicles correspondingly isolated from MDA-MB-231 cells. This RhoA/ROCK dependant signalling pathway that culminates in the formation of microvesicles in cancer cells, therefore, holds significant consequences for tumourigenesis. Phosphorylation of cofilin inhibits the actin-severing activity for the biogenesis of microvesicles in cancer cells [38]. The resulting elongation of actin filaments results in the formation of an *“actin ring”* structure which is essential for the maturation of EVs.

Another *in vivo* study showed that cofilin plays a role in promoting brain metastasis via the delivery of cancer-derived EVs that break down the blood-brain barrier (BBB) [74]. The brain-metastatic breast cancer cell lines used were MDA-MB-231-luc-D3H2LN, BMD2a and BMD2b cells. It was found that EVs derived from the cancer cells deliver miR-181c which promotes the destruction of BBB by downregulating its target gene 3-Phosphoinositide dependant Protein Kinase 1 (PDPK1), leading to the abnormal localisation of actin. Interestingly, PDPK1 degradation leads to the downregulation of phosphorylated cofilin and subsequently activates the cofilin-induced modulation of actin [74].

While concrete evidence for the involvement of cofilin-1 in EV mediated metastasis of TNBC is extremely limited, there have been several studies suggesting that cofilin-1 mediates metastasis of other aggressive cancers. Table 1 documents from the literature that cofilin-1 promotes cancer cell migration, is associated with poor prognosis, survival and may even promote cancer progression in a wide variety of cancer types. Specifically, in TNBC, there is evidence to show that cofilin-1 expression is correlated with cell proliferation, migration and metastasis. Therefore, evolving research into the mechanism of EV migration in patients with a diagnosis of TNBC holds great potential for unravelling the aggressiveness of TNBC. Notably, the true role of cofilin in the attenuation of TNBC metastasis via EVs is yet to be confirmed.

## Conclusion

Despite the evolving research into the role of EVs in cancer, few studies have focused solely on TNBC. The complex interaction of RhoA/ROCK/LIMK/Cofilin signalling networks as well as microRNA inhibition create a complex network and that cofilin-1 potentially plays a role in EV formation and establishment of premetastatic niches, therefore potentially promoting metastasis in TNBC patients. Similarly, factors transported by EVs such as miR-200b-3p and PDK1 may also have a role in this complex network. As the exact role of EVs in cancer progression emerges, in particular their complex role concerning cofilin-1 and actin, so too does the potential to target cofilin-1 for the treatment of metastatic TNBC. Further work into understanding the exact mechanism by which Cofilin-1 contributes to TNBC metastasis via the EVs has great potential to improve outcomes and prevent disease progression for patients.

## CRediT authorship contribution statement

**Jane Howard:** Conceptualization, Writing – original draft, Writing – review & editing. **Chia Yin Goh:** Conceptualization, Writing – review & editing. **Karolina Weiner Gorzel:** . **Michaela Higgins:** Writing – review & editing. **Amanda McCann:** Conceptualization, Writing – review & editing.

## Declaration of Competing Interest

None.
